# Protective Effects of (-)-Butaclamol Against Gentamicin-Induced Ototoxicity: In Vivo and In Vitro Approaches

**DOI:** 10.3390/ijms26094201

**Published:** 2025-04-28

**Authors:** Sumin Hong, Eunjung Han, Saemi Park, Kyungtae Hyun, Yunkyoung Lee, Hyun woo Baek, Hwee-Jin Kim, Yoon Chan Rah, June Choi

**Affiliations:** 1 Department of Otorhinolaryngology-Head and Neck Surgery, Korea University College of Medicine, Ansan Hospital, Ansan-si 15355, Republic of Korea; hongsumin0608@naver.com (S.H.); hoj7843@korea.ac.kr (E.H.); babydazzler@gmail.com (S.P.); pmik95@gmail.com (K.H.); leeyk83@gmail.com (Y.L.); bhw0810@korea.ac.kr (H.w.B.); hweejin2@gmail.com (H.-J.K.); ycrah@naver.com (Y.C.R.); 2 Laboratory of Otorhinolaryngology-Head & Neck Surgery, Graduate School of Medicine, Korea University, Seoul 02841, Republic of Korea; 3 Biomedical Research Center, Korea University College of Medicine, Ansan Hospital, Ansan-si 15355, Republic of Korea; 4 Zebrafish Translational Medical Research Center, Korea University, Seoul 02841, Republic of Korea; 5 Rare and Intractable Disease Research Group, Korea University, Ansan Hospital, Ansan-si 15355, Republic of Korea

**Keywords:** (-)-butaclamol, in vitro, in vivo, ototoxicity, gentamicin, hair cell

## Abstract

Gentamicin-induced ototoxicity leads to irreversible sensorineural hearing loss due to structural and functional damage to inner ear hair cells. In this study, we identified (-)-butaclamol as a potent protective agent against gentamicin-induced cytotoxicity through high-content screening (HCS) of a natural compound library. (-)-Butaclamol significantly enhanced cell viability in both HEI-OC1 cells and zebrafish neuromasts, demonstrating robust protection against gentamicin toxicity. Mechanistically, (-)-butaclamol inhibited intrinsic apoptosis, as evidenced by reduced TUNEL-positive cell counts and the downregulation of BAX and caspase-3, alongside the upregulation of BCL-2. Moreover, (-)-butaclamol activated key survival signaling pathways, including AKT/mTOR and ERK, while suppressing the inflammatory regulator NF-κB. Additional analyses revealed that (-)-butaclamol effectively mitigated oxidative stress and restored autophagic activity, as confirmed by CellROX and LysoTracker assays. Notably, TMRE staining showed that (-)-butaclamol preserved mitochondrial membrane potential in zebrafish hair cells, indicating mitochondrial protection. Collectively, these findings suggest that (-)-butaclamol exerts comprehensive cytoprotective effects against gentamicin-induced ototoxicity by modulating apoptosis, enhancing survival signaling, and restoring mitochondrial and cellular homeostasis. These results highlight the therapeutic potential of (-)-butaclamol and provide a foundation for future studies aimed at its clinical application.

## 1. Introduction

Ototoxic hearing loss is one of the side effects of various medications used to treat diseases. It occurs when inner ear function is compromised, leading to damage or destruction of structures, such as the cochlea and hair cells (HC), resulting in hearing loss [1,2]. Aminoglycosides are highly effective bactericidal agents frequently utilized in the treatment of infections caused by Gram-negative bacteria, such as sepsis and tuberculosis [3,4]. Among them, gentamicin is an aminoglycoside antibiotic that, when used in high doses or for extended periods, can cause direct damage to the HC located in the inner ear, leading to auditory impairments, such as hearing loss and tinnitus [5,6]. Gentamicin-induced ototoxicity is irreversible and characterized by sensorineural hearing loss, with mechanisms that include the induction of apoptosis in HC via reactive oxygen species (ROS) generation. Accumulation of ROS is a key mechanism underlying gentamicin-induced hearing loss [7,8]. Gentamicin disrupts the respiratory chain, promoting the production of mitochondrial ROS. This process involves the release of accumulated gentamicin into the cytoplasm, where it impacts mitochondrial function by activating the apoptotic pathway, inducing ROS generation, and depleting cellular ATP reserves [9,10,11]. Although various studies have been conducted on protecting the inner ear from ototoxicity, there is still a need to develop clinically safe and effective protective agents. Therefore, it is becoming increasingly important to explore new protective agents and conduct mechanistic studies focused on protecting HC in the inner ear.

In the current study, large-scale high-content screening (HCS) was conducted to identify potential protective agents against ototoxicity. Consequently, (-)-butaclamol demonstrated significant protective effects and was selected as a candidate drug for further analysis. (-)-Butaclamol is an antipsychotic agent that primarily functions as a dopamine D_2_ receptor antagonist [12]. This compound plays a crucial role in modulating dopaminergic pathways, which are vital for managing dopamine-related psychiatric disorders such as schizophrenia. Specifically, it exhibits a high affinity for dopamine D_2_ receptors, effectively inhibiting the action of dopamine by blocking its ability to activate these receptors, thereby exerting antipsychotic effects [12]. (-)-Butaclamol exists as two stereoisomers, (+) and (−), with (-)-butaclamol being the active form. The stereoisomer (-)-butaclamol is the active form with potent antipsychotic properties. Its high affinity for D_2_ receptors is pivotal in its effectiveness as an antipsychotic [13]. To date, (+)-butaclamol has been predominantly used to investigate the role of dopamine receptors or explore the mechanism of receptor antagonism in dopamine-related disease models. However, little is known about its protective role against ototoxicity. Therefore, the present study aimed to investigate the otoprotective effects of (-)-butaclamol and explore its potential as a therapeutic agent to prevent or treat ototoxicity.

Recent studies have highlighted the utility of HEI-OC1 cells and zebrafish models are valuable tools for investigating inner ear protection mechanisms and drug screening. The HEI-OC1 cell line is one of the most commonly used mouse auditory cell lines and is characterized by the maintenance of typical HC properties in culture [14,15]. It is widely used to study the mechanisms of ototoxicity in vitro, making it highly suitable for investigating these pathways. Consequently, HEI-OC1 cells are well suited for in vitro ototoxicity research [10,16]. The zebrafish (*Danio rerio*) is an established model organism for ototoxicity studies, offering the advantage of conserved vertebrate physiology. The lateral-line HC of zebrafish, which detects water vibrations, is structurally and functionally homologous to the HC of the human inner ear [17,18,19]. Zebrafish larvae models are useful for toxicological studies, providing a widely used system for investigating neuromast damage. Moreover, zebrafish respond rapidly to external environmental changes, offering a distinct advantage in in vivo studies for the quick assessment of the effects of ototoxic drugs [20,21]. These attributes make zebrafish a reliable model system for evaluating neuromast damage and related interventional factors induced by ototoxicity.

Recent studies have demonstrated the potential of utilizing both in vitro and in vivo models can be used in the research for protective agents, with particular emphasis on the HEI-OC1 cell line and zebrafish model as pivotal tools [10,18,22]. In this study, we aimed to explore substances that exhibit protective effects against gentamicin toxicity related to hearing loss using HEI-OC1 cells and zebrafish. This study can contribute to the development of novel therapeutic strategies to protect hearing loss from ototoxicity.

## 2. Results

### 2.1. (-)-Butaclamol Protects Against Gentamicin-Induced Ototoxicity in Both In Vitro and In Vivo Models

**Figure 1:**
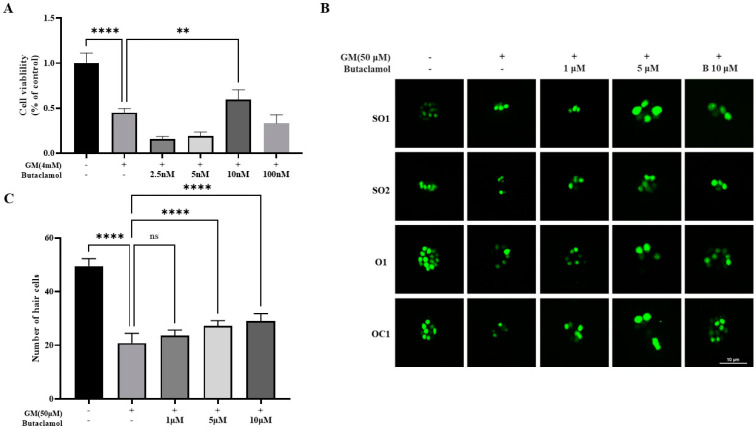
Assessment of cell viability using in vivo and in vitro models. (**A**) HEI-OC1 cell viability was evaluated using the CCK-8 assay in response to (-)-butaclamol treatment. HEI-OC1 cells (5 × 10^4^; cells/well in 96-well plates) were cultured for 24 h and then treated with gentamicin and (-)-butaclamol 48 h later. (**B**) The results indicate that (+)-butaclamol exerts a protective effect, as co-treatment with (-)-butaclamol and gentamicin mitigated ototoxicity in the hair cells located in the neuromasts of the supraorbital (SO1 and SO2), otic (O1), and occipital (OC1) lateral lines. At 5 days post-fertilization (dpf), wild-type zebrafish larvae were exposed to 50 μM gentamicin alone or in combination with (-)-butaclamol at concentrations of 1, 5, and 10 μM for 1 h. Following treatment, hair cells in the neuromasts were labeled using YO-PRO-1 staining (green) and subsequently examined under fluorescence microscopy. (20×; scale bar = 10 μm). (**C**) Quantification was performed to measure the protective effect of (-)-butaclamol against gentamicin-induced toxicity by assessing hair cell counts. Wild-type zebrafish larvae at 6 dpf (*n* = 30/group). Scale bar = 10 μm. (**** *p* < 0.0001, ** *p* < 0.01, ns, not significant; ANOVA). dpf, days post-fertilization; O, otic; OC, occipital; SO, supraorbital; P, posterior.

In the present study, we conducted a high-content screening (HCS) of a 1505-compound natural product library to identify compounds with protective effects against gentamicin-induced ototoxicity. Using HEI-OC1 cells treated with 4 mM gentamicin, we classified compounds as effective if they showed cell viability between 50% and 75% or higher relative to untreated controls. Several compounds exhibited protective potential; notably, butaclamol (compound **81**, Figure S1) demonstrated a particularly strong effect, achieving over 75% cell viability compared to the control. This high cell survival rate suggests that butaclamol may offer significant protective benefits. To further assess the efficacy of (-)-butaclamol, we proceeded with a CCK-8 assay using HEI-OC1 cells for more precise analysis. To optimize the gentamicin dosage, HEI-OC1 cells were treated for 24 h with various concentrations of gentamicin (4 mM) and (-)-butaclamol (2.5, 5, 10, or 100 nM), and cell viability was assessed using the CCK-8 assay. The results indicated that co-treatment with 10 nM of (-)-butaclamol exhibited a protective effect against gentamicin-induced cytotoxicity (Figure 1A). Subsequently, to further validate the efficacy of the candidate protective agents in vivo, zebrafish were used. Zebrafish neuromast cells share structural and biological similarities with human auditory cells [23]. Therefore, the protective effect of butaclamol was analyzed using the supraorbital (SO1 and SO2), otic (O1), and occipital (OC1) lateral lines, and the total number of HC was quantified. After treating zebrafish larvae with combinations of gentamicin at concentrations of 1, 5, and 10 µM along with (-)-butaclamol, microscopic observations revealed a significant reduction in HC counts in the GM group compared with that in the ㄴ control group. In contrast, the (-)-butaclamol-treated group exhibited a tendency for a greater number of HC to be preserved compared with that of the GM treatment (Figure 1B). Statistically, (-)-butaclamol demonstrated protective effects against 10 µM gentamicin-induced toxicity (Figure 1C).

### 2.2. (-)-Butaclamol Reduces Gentamicin-Induced HC Apoptosis

**Figure 2:**
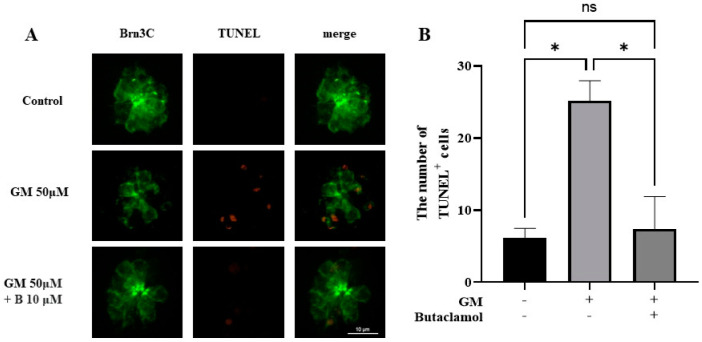
TUNEL assay for detecting apoptosis in hair cells of zebrafish. (**A**) Representative images of TUNEL staining (red) in the otic (O1) neuromast of 5 dpf transgenic zebrafish larvae (Brn3c:EGFP) (green). Larvae were exposed to 50 μM gentamicin (GM) or co-treated with 50 μM gentamicin and 10 μM (-)-butaclamol (GM+B) for 1 h. (-)-Butaclamol treatment reduced gentamicin-induced apoptosis. (20×; scale bar = 10 μm). (**B**) Quantification of the average number of TUNEL-positive cells in four neuromasts (SO1, SO2, O1, and OC1) demonstrates a significant increase in apoptosis following gentamicin treatment, which was attenuated by (-)-butaclamol co-treatment (*n* = 6 per group; * *p* < 0.05; ns, not significant). GM, gentamicin; B, butaclamol.

Gentamicin induces HC death via apoptosis, which contributes to hearing loss [24]. To investigate whether (-)-butaclamol exhibits a protective effect against gentamicin-induced cell death, a TUNEL assay was used. Based on the results from prior HC count analyses, the experiment was conducted with 10 μM of (-)-butaclamol, which showed the highest protective efficacy. Microscopic observations revealed that the GM group had a higher number of TUNEL-positive cells than the control group. However, when both gentamicin and (-)-butaclamol were co-administered, the number of TUNEL-positive cells was reduced compared to that in the GM group (Figure 2A). Upon quantifying the total number of TUNEL-positive cells, no significant differences were observed between the GM+B and control groups. However, a significant reduction in the number of TUNEL-positive cells was observed in the GM+B group compared to that in the GM group (Figure 2B). Treatment with (-)-butaclamol significantly decreased the number of TUNEL-positive HC, indicating that apoptosis was inhibited.

### 2.3. (-)-Butaclamol Attenuates Gentamicin-Induced Ototoxicity by Inhibiting Intrinsic Apoptotic Signaling

Apoptosis, or programmed cell death, is primarily regulated by the modulation of proteins involved in the formation of death-inducing signaling complexes. The TUNEL assay revealed a reduction in apoptosis following (-)-butaclamol treatment. Given the potential role of (-)-butaclamol as a protective agent against gentamicin-induced cytotoxicity, we investigated whether (-)-butaclamol exerted this protective effect by inhibiting apoptosis. To assess this, we performed Western blotting using markers associated with apoptosis.

**Figure 3:**
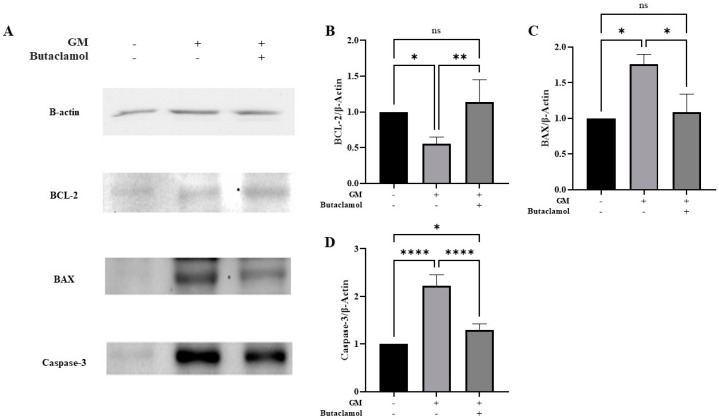
(-)-Butaclamol modulates intrinsic apoptotic signaling in gentamicin-treated HEI-OC1 cells. HEI-OC1 cells were treated with 4 mM gentamicin alone or co-treated with 4 mM gentamicin and 10 nM (-)-butaclamol for 48 h. Western blotting was performed to assess the expression of intrinsic apoptotic markers, including BCL-2, BAX, and caspase-3. (**A**) Representative Western blot images for BCL-2, BAX, caspase-3, and β-actin as a loading control. (**B**) Quantification of BCL-2 expression shows that gentamicin reduced BCL-2 levels, whereas (-)-butaclamol co-treatment restored BCL-2 expression to levels comparable to the control. (**C**) BAX expression was significantly increased by gentamicin treatment and reduced by (-)-butaclamol to near-control levels. (**D**) Caspase-3 activation was induced by gentamicin but significantly suppressed in the co-treatment group. (* *p* < 0.05; ** *p* < 0.01; **** *p* < 0.0001; ns, not significant; one-way ANOVA). GM, gentamicin; B, butaclamol.

We specifically examined the expression levels of Bax and Bcl-2, which are key regulators of the apoptotic pathway. To further investigate the apoptotic mechanisms underlying the protective effects of (-)-butaclamol, we examined the expression levels of key apoptotic markers, including BCL-2, BAX, and caspase-3 (Figure 3A). As shown in Figure 3B, BCL-2 expression was significantly decreased in the gentamicin-treated group, whereas co-treatment with (-)-butaclamol restored BCL-2 levels to a level comparable with that of the control group. In contrast, the expression of BAX was markedly increased in the gentamicin group but was significantly reduced in the group co-treated with (-)-butaclamol, again approaching control levels (Figure 3C). Furthermore, caspase-3, a major executioner of apoptosis, was strongly activated by gentamicin, and this upregulation was significantly attenuated by (-)-butaclamol co-treatment (Figure 3D). These findings suggest that the protective effects of (-)-butaclamol involve inhibition of the intrinsic apoptotic pathway via modulation of BCL-2 and BAX expression and suppression of caspase-3 activation.

### 2.4. (-)-Butaclamol Enhances Cell Survival Through ERK and AKT/mTOR Pathway Activation in Gentamicin-Induced Ototoxicity

**Figure 4:**
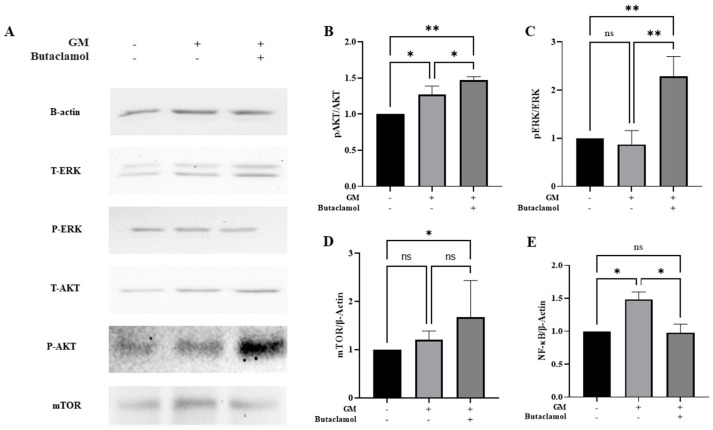
Mechanistic insights into (-)-butaclamol protective effects. HEI-OC1 cells were treated with 4 mM gentamicin alone or co-treated with 4 mM gentamicin and 10 nM (-)-butaclamol for 48 h to explore the molecular mechanisms underlying the protective effects of (+)-butaclamol. Western blotting was performed on 40 µg of total protein lysates using specific primary antibodies. (**A**–**E**) Representative blots and quantification of protein expression levels: pAKT/AKT (b), pERK/ERK (c), mTOR (**D**), and NF-κB (**E**). Significant upregulation of survival signaling markers was observed following (-)-butaclamol treatment. (* *p* < 0.05; ** *p* < 0.01; ns, not significant; ANOVA). GM, gentamicin; B, butaclamol.

No significant changes were observed in the apoptosis-related proteins; therefore, we investigated alternative protective mechanisms. The D2 dopamine receptor is closely associated with the PI3K/AKT/mTOR pathway [12,25]. Given that (-)-butaclamol is a D2 receptor antagonist, we investigated whether it influences this pathway. Our results demonstrated that the levels of AKT and ERK proteins were significantly increased when (-)-butaclamol was co-administered with gentamicin, compared to that with gentamicin treatment alone (Figure 4A–C). Additionally, a marked increase in AKT and ERK levels was observed in the (-)-butaclamol-treated group compared with that in the control group. mTOR, a key regulator of cell growth and survival, also exhibited enhanced activation, as indicated by an increase in *p*-AKT/AKT levels (Figure 4A,D). Furthermore, gentamicin-induced ototoxicity is partly mediated by inflammation-induced cell death; therefore, we examined NF-kB, a major regulator of inflammatory responses. (-)-Butaclamol significantly reduced NF-kB levels compared to that with gentamicin alone (Figure 4A,E), suggesting that it mitigates ototoxicity by activating survival pathways (AKT/mTOR and ERK) and suppressing inflammatory responses (via NF-kB inhibition). In summary, our findings imply that (-)-butaclamol enhances cell survival mechanisms primarily via coordinated activation of the ERK and AKT/mTOR signaling pathways rather than by suppressing apoptosis.

### 2.5. (-)-Butaclamol Exhibits a Significant Inhibitory Effect on ROS Production

**Figure 5:**
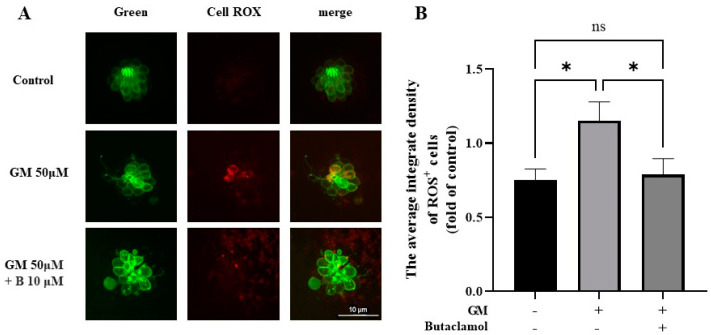
CellROX assay for detecting ROS in zebrafish hair cells. (**A**) Representative images of CellROX staining (red) in 5 days post-fertilization (dpf) Brn3c:EGFP zebrafish larvae (green) treated with control medium, gentamicin (GM 50 μM), or gentamicin + (-)-butaclamol (GM 50 μM + B 10 μM). Larvae were exposed to the respective treatments for 1 h, followed by staining with 5 μM CellROX reagent for 30 min in the dark. Images were taken from neuromasts using a fluorescence microscope (20×; scale bar = 10 μm). (**B**) Quantification of the average integrated density of CellROX fluorescence across four neuromasts (SO1, SO2, O1, and OC1) shows that gentamicin significantly increased ROS levels, whereas co-treatment with (-)-butaclamol significantly reduced ROS production compared to the gentamicin-only group (*n* = 6 per group; * *p* < 0.05; ns, not significant; ANOVA). GM, gentamicin; B, butaclamol.

Multiple studies have indicated that gentamicin induces the production of ROS in the inner ear, which can damage sensory cells, including HCs, ultimately leading to cell death [24]. Previous research has confirmed the activation of the AKT pathway, which regulates oxidative stress [26]. Thus, we used the CellROX assay to investigate whether (-)-butaclamol could reduce gentamicin-induced ROS production. In the GM group, a significant ROS-positive response was observed in HC; however, no such response was detected in the control group or GM+B group. Notably, the HC treated with (-)-butaclamol exhibited a pattern similar to that of the control group (Figure 5A). To assess oxidative stress in hair cells, we measured intracellular ROS levels using CellROX staining in 5 dpf zebrafish larvae. As shown in Figure 5B, gentamicin treatment significantly increased ROS production compared to the control group, consistent with its known pro-oxidant effects. Notably, co-treatment with (+)-butaclamol significantly reduced ROS levels relative to the gentamicin-only group, suggesting that (-)-butaclamol attenuates gentamicin-induced oxidative stress.

### 2.6. Autophagy Mediated by (-)-Butaclamol Protects HCs from Gentamicin-Induced Damage

**Figure 6:**
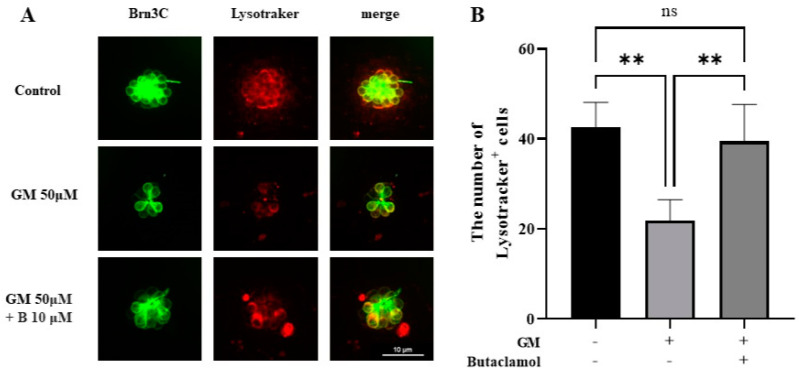
Increased autophagy in hair cells after (-)-butaclamol + gentamicin co-treatment. (**A**) Representative images of LysoTracker Red DND-99 staining in 5 dpf transgenic zebrafish larvae (Brn3c:EGFP) from the control, gentamicin-treated (GM 50 μM), and gentamicin + (-)-butaclamol co-treated (GM 50 μM + B 10 μM) groups. Larvae were incubated in LysoTracker solution for 30 min in the dark following drug exposure and washing steps (20×; scale bar = 10 μm). (**B**) Quantification of the average number of LysoTracker-positive neuromasts (SO1, SO2, O1, and OC1) shows a significant reduction in autophagic activity in the gentamicin group, which was significantly restored by (-)-butaclamol co-treatment (** *p* < 0.01; ns, not significant; ANOVA; *n* = 6 per group). GM, gentamicin; B, butaclamol.

Autophagy is a crucial factor in the response to cellular stress and has potential pathological implications in neurodegenerative diseases and aging [27]. Autophagy is linked to hearing loss through its association with ROS during ototoxicity [28]. Therefore, we aimed to analyze whether gentamicin-induced ototoxicity is related to autophagy using LysoTracker staining. In the control and 50 µM GM+B group, we observed a significant increase in autophagic activity compared with that in the GM group (Figure 6A). Although no statistically significant changes were observed in the group treated with protective agents compared with those in the GM group, LysoTracker staining levels showed a pattern similar to that of the control group, indicating an increase in autophagic activity (Figure 6B). Autophagy regulates both cell survival and death, depending on the context. Treatment with (-)-butaclamol appears to promote autophagy in a manner that protects hair cells from gentamicin-induced damage [29,30].

### 2.7. (-)-Butaclamol Preserves Mitochondrial Membrane Potential in Gentamicin-Treated Zebrafish Hair Cells

**Figure 7:**
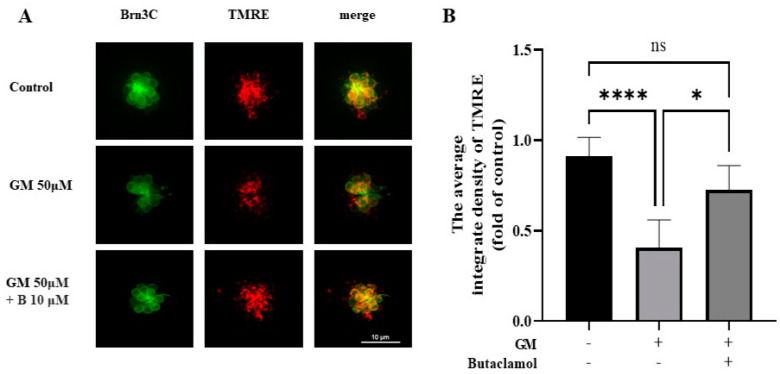
Protective effect of (-)-butaclamol on mitochondrial membrane potential in gentamicin-treated zebrafish hair cells. (**A**) Representative images of TMRE staining in the control, gentamicin-treated (GM 50 μM), and (-)-butaclamol co-treated (GM 50 μM + B 10 μM) groups in 5 days post-fertilization (dpf) Brn3c:EGFP zebrafish larvae. Larvae were treated with the respective drugs for 1 h, rinsed, and then incubated with 250 nM TMRE for 30 min prior to imaging of the neuromasts. (20×; scale bar = 10 μm). (**B**) Quantification of the average integrated density of TMRE fluorescence in four neuromasts (SO1, SO2, O1, and OC1) revealed that gentamicin significantly decreased mitochondrial membrane potential, while co-treatment with (-)-butaclamol significantly restored TMRE intensity to near-control levels, indicating its protective role in preserving mitochondrial function (* *p* < 0.05; **** *p* < 0.0001; ns, not significant; ANOVA).

Mitochondria, which play a central role as key regulators of HC physiology by providing energy in the form of ATP, are typically analyzed using TMRE as a marker to evaluate mitochondrial membrane potential and the impact of ototoxic drugs, such as gentamicin, on cellular health [31]. TMRE staining was performed to investigate the mitochondrial dysfunction in HCs treated with gentamicin and protective agents. To evaluate mitochondrial membrane potential as an indicator of mitochondrial function, TMRE staining was performed in 5 dpf Brn3c:EGFP zebrafish larvae. As shown in Figure 7A, TMRE fluorescence intensity markedly decreased in the gentamicin-treated group, indicating significant mitochondrial depolarization. In contrast, the group co-treated with (-)-butaclamol exhibited a significant restoration of TMRE signal compared to the gentamicin-only group (Figure 7B). These results suggest that (-)-butaclamol exerts a protective effect on mitochondrial function by preventing gentamicin-induced loss of mitochondrial membrane potential.

## 3. Discussion

Gentamicin-induced ototoxicity is characterized by irreversible sensorineural hearing loss resulting from direct structural damage to the inner ear. Once the inner ear is damaged by ototoxicity, recovery is not possible, and no effective treatment has been developed [4,32]. In this study, we conducted drug screening using HCS and identified (-)-butaclamol as a potential candidate for protection against gentamicin-induced ototoxicity. The aim of this study was to investigate the protective effects of (-)-butaclamol and explore the underlying mechanisms, with the goal of preventing gentamicin-induced ototoxicity and assessing its potential as a therapeutic agent. Our findings demonstrate that while (-)-butaclamol protects against gentamicin-induced apoptosis, its protective role is mediated by the promotion of cell survival signaling pathways rather than by direct inhibition of apoptosis. This suggests that (-)-butaclamol is a promising potential therapeutic agent for protecting HC from gentamicin-induced damage. The zebrafish model, which shares structural and functional similarities with mammalian auditory systems, provided an ideal platform to evaluate the in vivo protective effects of (-)-butaclamol. Alongside HEI-OC1 cell-based assays, our results consistently showed improved cell viability and preserved hair cell integrity, establishing a strong foundation for future translational research.

In the current study, gentamicin treatment resulted in significant cell death in both HC of zebrafish and HEI-OC1 cells, as evidenced by the increased TUNEL-positive staining, which indicates apoptosis. The protective role of (-)-butaclamol was demonstrated by its ability to enhance cell viability and reduce the number of TUNEL-positive cells, suggesting that (-)-butaclamol mitigates apoptosis. Western blot analyses further confirmed this effect by showing a significant upregulation of BCL-2 and a downregulation of BAX and caspase-3 in the (-)-butaclamol-treated group compared to the gentamicin group. These results indicate that (-)-butaclamol suppresses intrinsic apoptotic signaling, supporting its role as an anti-apoptotic agent. This new evidence strengthens the conclusion that the reduction in cell death is not merely a trend but is mechanistically underpinned by the modulation of core apoptotic regulators.

The BCL-2 family of proteins plays a pivotal role in mitochondrial-mediated apoptosis by controlling the release of cytochrome c and the activation of downstream caspases [33,34]. By restoring BCL-2 and downregulating BAX, (-)-butaclamol likely stabilizes the mitochondrial membrane and inhibits apoptosome formation [35,36]. These actions ultimately suppress caspase-3 activation, a final effector of the intrinsic apoptotic pathway, thereby preventing irreversible cell death in cochlear-like cells [37].

In our findings, we observed an increase in the levels of pAKT/AKT, pERK/ERK, and mTOR, while the immune modulator NF-κB was significantly reduced. ERK signaling is commonly associated with the promotion of cell death, particularly in cases of ototoxicity, where elevated ERK activity is known to exacerbate cell death. However, our study suggested a shift in the role of ERK from a pro-apoptotic to a pro-survival signaling pathway. The role of ERK in determining cell survival or death is highly context-dependent, with multiple studies indicating its dual functions, particularly in ototoxicity and related cellular stress models. Studies on neuronal cells have shown that ERK activation can enhance the phosphorylation of AKT and ERK1/2, promoting cell survival by inhibiting apoptotic pathways, such as the cleavage of caspase-9 and caspase-3 [38]. Moreover, in certain cancer models, ERK is described as a “double-edged sword”, capable of promoting apoptosis under specific conditions but supporting cell proliferation and survival when appropriately regulated [39]. This dynamic role of ERK aligns with our findings, where (-)-butaclamol appeared to modulate ERK signaling, shifting it toward promoting cell survival. This modulation could explain the observed increase in ERK activation without concurrent cell death, indicating that (-)-butaclamol may redirect ERK signaling to favor survival over apoptosis during gentamicin-induced damage. Notably, AKT and ERK convergence on mTOR activation suggests that (-)-butaclamol orchestrates a broader pro-survival network. Consistently, previous research indicates that ERK signaling can shift from promoting cell death to supporting cellular repair and survival, depending on its regulatory context [40]. In therapeutic or protective contexts, ERK activation promotes cell proliferation and recovery, ultimately contributing to cell survival. Our results imply that (-)-butaclamol has a protective role, providing further insights into its mechanism of action against gentamicin-induced ototoxicity.

In this study, we elucidated the mechanisms by which (-)-butaclamol contributes to cellular survival by normalizing autophagy and reducing oxidative stress using CellROX and LysoTracker data. CellROX data indicated that (-)-butaclamol treatment led to a significant decrease in intracellular oxidative stress, demonstrating its ability to modulate oxidative stress responses and reduce cellular damage. This aligns with previous findings, where the activation of the AKT pathway under oxidative stress plays a pivotal role in mitigating ROS accumulation, thereby promoting recovery from cellular injury [41].

Such mechanisms highlight the importance of the AKT/mTOR pathway in oxidative stress regulation, as it helps reduce cellular sensitivity to oxidative stimuli, as demonstrated in several studies [42]. Gentamicin-induced ROS production is a known contributor to HC apoptosis, often acting upstream of mitochondrial collapse and inflammation. By suppressing ROS, (-)-butaclamol not only reduces oxidative burden but also likely prevents secondary cell death cascades. Moreover, the LysoTracker data revealed the normalization of lysosomal activity, suggesting that (-)-butaclamol supports the autophagic pathway. Autophagy is essential for maintaining cellular homeostasis, particularly for the removal of damaged proteins and organelles. The activation of autophagy in HC of zebrafish and HEI-OC1 cell lines not only reduces ROS levels but also promotes cell survival [43]. Furthermore, proper autophagic flux is crucial for mitochondrial quality control, and its dysregulation is often linked to degenerative auditory diseases. The restoration of normal autophagic processes is crucial for cell survival under stress, as autophagy plays a protective role by maintaining cellular integrity. Consistently, previous studies indicated that the balance between oxidative stress and autophagy is mediated by the mTOR pathway, which regulates mitochondrial ROS and lysosomal function [42]. Therefore, the activation of the AKT/mTOR pathway by (-)-butaclamol not only reduces oxidative stress but also restores autophagy, creating a robust cellular defense mechanism. These findings suggested that (-)-butaclamol could serve as a potential therapeutic agent by targeting both oxidative stress and autophagy to promote cellular survival. The results from the CellROX and LysoTracker data provide strong evidence that the protective effects of (-)-butaclamol extend beyond simple oxidative stress suppression, offering a multifaceted approach to enhancing cell viability.

Furthermore, TMRE staining confirmed that (-)-butaclamol preserves mitochondrial membrane potential in zebrafish hair cells exposed to gentamicin. Gentamicin-induced mitochondrial depolarization is a hallmark of early apoptotic signaling and cellular energy collapse. Our data demonstrated that TMRE intensity was significantly restored in the (-)-butaclamol-treated group, suggesting that mitochondrial integrity is preserved. This further reinforces the notion that (-)-butaclamol not only suppresses cytotoxic cascades but also maintains essential organelle functions necessary for cell survival.

Mitochondrial preservation is particularly critical in ototoxic injury, where the early loss of membrane potential leads to ATP depletion, cytochrome c release, and caspase activation. By preventing these initial disruptions, (-)-butaclamol may interrupt upstream apoptotic signals and maintain metabolic activity essential for long-term cellular integrity. The combined modulation of apoptosis, oxidative stress, autophagy, and mitochondrial function positions (-)-butaclamol as a promising multifunctional therapeutic candidate for the prevention of aminoglycoside-induced auditory degeneration.

**Figure 8:**
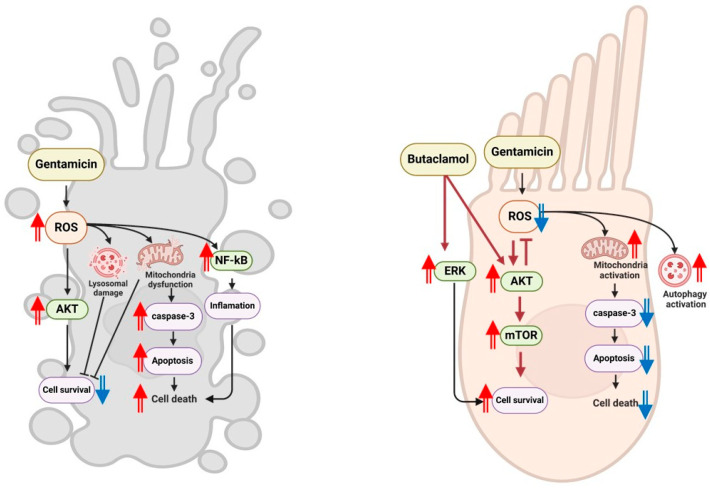
Schematic summary of (-)-butaclamol against gentamicin-induced ototoxicity. The schematic diagram illustrates that (-)-butaclamol has a protective role against gentamicin-induced ototoxicity by enhancing cell survival, mainly by regulation of the AKT/mTOR pathway, the reduction of oxidative stress, and the restoration of autophagic balance. Statistically significant increases or decreases are indicated by two solid arrows (red for increase, blue for decrease).

In summary, our results demonstrate that (-)-butaclamol provides multifaceted cytoprotective effects against gentamicin-induced ototoxicity by modulating intrinsic apoptotic pathways, activating pro-survival signaling (AKT/mTOR and ERK), reducing oxidative stress, restoring autophagic activity, and preserving mitochondrial membrane potential. These effects were validated in both in vitro and in vivo models, supporting the translational potential of (-)-butaclamol. Notably, TMRE analysis revealed that (-)-butaclamol maintains mitochondrial integrity, a key determinant of cellular energy status and survival. The dual activation of AKT and ERK, alongside NF-κB suppression, suggests a coordinated pro-survival response. These findings collectively propose (-)-butaclamol as a promising therapeutic agent for preventing aminoglycoside-induced hair cell degeneration. Future studies should evaluate its clinical applicability in combination with mitochondrial-targeted antioxidants or explore pharmacological modifications to optimize its efficacy in restoring organelle-specific function during ototoxic insult (Figure 8).

## 4. Materials and Methods

### 4.1. In Vitro Drug Screening

The high-content screening (HCS) was conducted using the Live/Dead Assay Kit from EarlyTox (EarlyTox, Molecular Devices, San Jose, CA, USA; Cat. No. R8340), following the manufacturer’s protocol. Cells were plated in Greiner 384-well microplates and screened using the ImageXpress Micro 4 high-content screening (HCS) system (Molecular Devices, San Jose, CA, USA). to analyze the drug library. A natural product library (MedChemExpress, Monmouth Junction, NJ, USA) consisting of 1505 compounds was provided by the Korea Chemical Bank (www.chembank.org) and screened. Following the initial screening, (-)-butaclamol showed efficacy and was purchased and used in further studies. In this study, gentamicin (Gibco Co., Ltd., New York, NY, USA, Cat. No. 15750060) was utilized and stored at room temperature. Butaclamol from AmBeed, Inc. (Arlington Heights, IL, USA, CAS No. 2693-46-1) was dissolved in dimethyl sulfoxide (DMSO, Sigma-Aldrich, Cat. No. D2650, St. Louis, MO, USA) and stored in the dark at 2–8 °C.

### 4.2. In Vitro Assay

HEI-OC1 cells, the auditory organ of a transgenic mouse cell line, were used in this study and were provided by F. Kalinec (House Ear Institute, Los Angeles, CA, USA). HEI-OC1 cells were incubated at 33 °C in 10% CO_2_ and cultured in Dulbecco’s modified Eagle medium (DMEM; Corning, NY, USA, Cat. No. 10-013-CV) with 10% fetal bovine serum (FBS, Gibco, Cat. No. 16000044) [16]. HEI-OC1 cells were treated with gentamicin (4 mM) and used for CCK-8 (CELL COUNTING KIT-8, Dojindo Laboratories, Kumamoto, Japan, Cat. No. CK04-11) and Western blotting. HEI-OC1 cells (1 × 10^4^ cells/well in 6-well plates) were cultured for 24 h and then treated with gentamicin and (-)-butaclamol 48 h later. The 4 mM gentamicin concentration was determined to be the concentration required for 50% inhibition, also known as the half-maximal inhibitory concentration (IC50). In addition, 2.5, 5, 10, and 100 nM (-)-butaclamol were dissolved in the culture medium and treated with a 48 h incubation period. Cell viability was assessed using the CCK-8 kit (Dojindo Laboratories, Kumamoto, Japan).

### 4.3. Zebrafish Housing

Wild-type zebrafish and transgenic zebrafish (Brn3c:EGFP) were bred in the zebrafish facility at the Korea University Ansan Hospital at 28.5 °C. Following pair mating, zebrafish embryos were maintained in a standard embryo medium (15 mM NaCl, 0.5 mM KCl, 1 mM CaCl_2_, 1 mM MgSO_4_, 0.15 mM KH_2_PO_4_, 0.05 mM NH_2_PO_4_, and 0.7 mM Na-HCO_3_) of the Petri dish. Zebrafish larvae at five days post-fertilization (dpf) were utilized in all subsequent experiments. This study was approved by the Korea University Institutional Animal Care and Use Committee (approval no. KOREA-2024-0154). All experiments were performed in accordance with the guidelines of the Animal Care Ethics Committee of the Korea University Medical Centre and the National Institute of Health guidelines.

### 4.4. Counting Hair Cells in Neuromasts

At 5 dpf, wild-type zebrafish larvae were treated with 50 μM of gentamicin alone and combined doses of 1, 5, and 10 μM (-)-butaclamol for 1 h. The gentamicin concentration was determined as previously described, utilizing the 50% lethal concentration values established to induce HC death in zebrafish. Embryos were then rinsed several times with embryo medium, anesthetized with tricaine, and observed under a fluorescence microscope (Eclipse Ni-U, Nikon, Tokyo, Japan) to examine the neuromasts of the supraorbital (SO1 and SO2), otic (O1), and occipital (OC1) lateral lines. In both experimental and control conditions, the total number of HCs in the SO1, SO2, O1, and OC1 neuromasts of each zebrafish was counted.

### 4.5. Terminal Deoxynucleotidyltransferase (TdT)-Mediated dUTP-Biotin Nick End Labeling (TUNEL) Assay

To assess HC apoptosis, a TUNEL assay was performed using an in situ cell detection kit (Roche Molecular Biochemicals, Mannheim, Germany, Cat. No. 11684795910). In this study, 5 dpf transgenic zebrafish larvae (Brn3c:EGFP) were used. To investigate the interaction between gentamicin and (-)-butaclamol, the experiment was conducted with three groups: the control (wild-type), gentamicin-treated (GM; 50 μM), and gentamicin + (-)-butaclamol-treated groups (GM+B; 10 μM). The zebrafish were exposed to media containing 50 μM of gentamicin and 10 μM of (-)-butaclamol for 1 h, followed by fixation in 4% paraformaldehyde. Subsequently, 50 μL of the TUNEL reaction mixture (TdT and fluorescein-dUTP) was applied for 1 h at 37 °C, and the samples were observed under a fluorescence microscope. The neuromasts (SO1, SO2, O1, and OC1) of each zebrafish were examined, and the total number of TUNEL-positive cells was counted to compare the apoptosis induced by gentamicin and (-)-butaclamol.

### 4.6. Quantifying ROS Levels in Hair Cells

To investigate ROS generation in zebrafish HCs and its effects, CellROX™ Deep Red reagent (Invitrogen, Carlsbad, CA, USA, Cat. No. C10422) was used in 5 dpf transgenic zebrafish larvae. The larvae were divided into three groups: the control (wild-type), GM (50 μM), and GM+B-treated groups (10 μM). Each group was exposed to media containing the respective treatment, followed by 5 µM CellROX™ Deep Red reagent in the dark. After washing with phosphate-buffered saline, the samples were fixed with 4% paraformaldehyde. Fluorescence intensity was observed under a fluorescence microscope in four neuromasts (SO1, SO2, O1, and OC1). The average intensity for each group was calculated, and ROS levels were compared among the control, GM, and GM+B-treated groups.

### 4.7. Quantifying Mitochondrial Transmembrane Potential (ΔΨm) Loss in Hair Cells

Tetramethylrhodamine ethyl ester (TMRE) labeling (Invitrogen, Cat. No. T669, Waltham, MA, USA) was used to assess mitochondrial membrane potential loss. Transgenic zebrafish larvae at 5 dpf were divided into three groups: the control (wild-type), GM (50 μM), and GM+B-treated groups (10 μM). Following the administration of the pharmacological agents, the larvae were rinsed with embryo media and incubated with 250 nM TMRE for 30 min. The samples were washed multiple times, anesthetized with tricaine, and examined using a fluorescence microscope to observe the neuromasts of the zebrafish. Integrated density measurements were performed for each group, and the average integrated density of the four neuromasts was calculated for comparative analysis.

### 4.8. Qualitative Analysis of Autophagy Activation

Autophagic activity in HCs was observed using LysoTracker Red D-99 (Invitrogen, Cat. No. L7528). At 5 dpf, transgenic zebrafish larvae from the control (wild-type), GM (50 μM), and GM+B-treated groups (10 μM) underwent triple-washing with embryo media. Subsequently, the larvae were immersed in a solution of LysoTracker Red DND-99 diluted in the embryo media and incubated in the dark for 30 min. The number of neuromasts labeled with LysoTracker was counted for each group, and the mean number of labeled neuromasts was calculated for comparative analysis.

### 4.9. Western Blotting

HEI-OC1 cells were cultured and treated with gentamicin and (-)-butaclamol. The cells were then lysed using lysis buffer, and 40 µg of total protein was denatured by heating at 100 °C for 5 min before being loaded onto a 10% SDS-PAGE gel. After electrophoresis, the proteins were transferred onto apolyvinylidene fluoride (PVDF) membranes and blocked using blocking buffer. Subsequently, the membrane was incubated with primary antibodies against AKT (Cell Signaling Technology, Danvers, MA, USA, Cat. No. 9272), ERK (Cell Signaling Technology, Cat. No. 4695), mTOR (Cell Signaling Technology, Cat. No. 2983), BCL-2 (Abcam, Cat. No. ab182858), BAX (Abcam, Cat. No. ab32503), caspase-3 (Cell Signaling Technology, Cat. No. 9662), and NF-κB (Cell Signaling Technology, Danvers, MA, USA, Cat. No. 8242). Detection was performed using chemiluminescence with the Thermo Fisher Scientific Pierce ECL Western Blotting Substrate (Thermo Fisher Scientific, Waltham, MA, USA, Cat. No. 32106).

### 4.10. Statistical Analysis

To perform statistical analyses among multiple groups, we utilized appropriate methods, either one-way analysis of variance (ANOVA) for normally distributed groups or the Kruskal–Wallis test for groups with non-normal distributions. Tukey’s honest significant difference test and Bonferroni correction were applied for post hoc analyses, as appropriate. Statistical significance was set at *p* < 0.05. All statistical analyses were performed using Prism 9 for Windows (La Jolla, CA, USA), and all data are shown as means ± standard errors.

## 5. Conclusions

Gentamicin-induced ototoxicity leads to irreversible sensorineural hearing loss due to structural and functional damage to inner ear hair cells. In this study, we identified (+)-butaclamol as a potent protective agent against gentamicin-induced cytotoxicity through high-content screening (HCS) of a natural compound library. (-)-Butaclamol significantly enhanced cell viability in both HEI-OC1 cells and zebrafish neuromasts, demonstrating robust protection against gentamicin toxicity.

Mechanistically, (-)-butaclamol inhibited intrinsic apoptosis, as evidenced by reduced TUNEL-positive cell counts and the downregulation of BAX and caspase-3, alongside the upregulation of BCL-2. Moreover, (-)-butaclamol activated key survival signaling pathways, including AKT/mTOR and ERK, while suppressing the inflammatory regulator NF-κB. Additional analyses revealed that (-)-butaclamol effectively mitigated oxidative stress and restored autophagic activity, as confirmed by CellROX and LysoTracker assays. Notably, TMRE staining showed that (-)-butaclamol preserved mitochondrial membrane potential in zebrafish hair cells, indicating mitochondrial protection. Collectively, our findings suggest that (-)-butaclamol exerts comprehensive cytoprotective effects against gentamicin-induced ototoxicity by modulating apoptosis, enhancing survival signaling, and restoring mitochondrial and cellular homeostasis. These results highlight the therapeutic potential of (-)-butaclamol and provide a foundation for future studies aimed at its clinical application.

## Data Availability

The datasets generated and/or analyzed during the current study are available from the corresponding author upon reasonable request.

## Supplementary Materials

The following supporting information can be downloaded at https://www.mdpi.com/article/10.3390/ijms26094201/s1.
