# Supplementary material for: Protective Effects of (-)-Butaclamol Against Gentamicin-Induced Ototoxicity: In Vivo and In Vitro Approaches
Source: Int J Mol Sci. 2025 Apr 28;26(9):4201. doi: 10.3390/ijms26094201 (PMC12071943; doi:10.3390/ijms26094201)
Supplement: Supplementary file 1 [file ijms-26-04201-s001.zip › ijms-3519899-supplementary.pdf]

International Journal of *Molecular Sciences*

MDPI

Supplementary Material

Protective Effects of (-)-Butaclamol Against Gentamicin-Induced Ototoxicity: In Vivo and In Vitro Approaches

Sumin Hong, Eunjung Han, Saemi Park, Kyungtae Hyun, Yunkyoung Lee, Hyun woo Baek, Hwee-Jin Kim, Yoon Chan Rah and June Choi

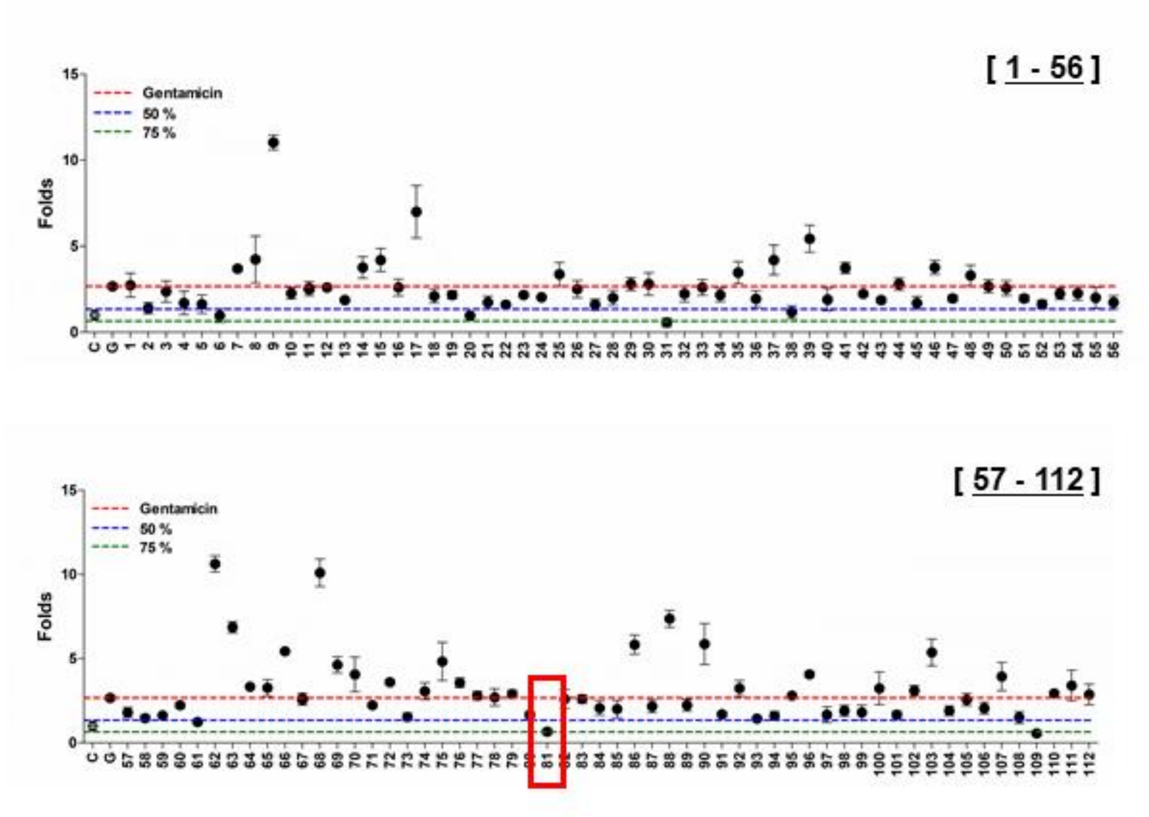

Figure S1. High-content screening (HCS) analysis using HEI-OC1 cells.

In total, 1,505 compounds were screened, among which (+)-butaclamol (compound 81, highlighted in red) was identified as the lead candidate. (+)-Butaclamol reduced cell viability by 75%. The full data are not disclosed.
